# Detection of Real-World Trips in At-Fall Risk Community Dwelling Older Adults Using Wearable Sensors

**DOI:** 10.3389/fmed.2020.00514

**Published:** 2020-09-02

**Authors:** Shirley Handelzalts, Neil B. Alexander, Nicholas Mastruserio, Linda V. Nyquist, Debra M. Strasburg, Lauro V. Ojeda

**Affiliations:** ^1^Division of Geriatric and Palliative Medicine, Department of Internal Medicine, University of Michigan, Ann Arbor, MI, United States; ^2^Department of Physical Therapy, Faculty of Health Sciences, Ben-Gurion University of the Negev, Beer-Sheva, Israel; ^3^Department of Physical Therapy, Loewenstein Rehabilitation Hospital, Ra'anana, Israel; ^4^VA Ann Arbor Health Care System Geriatrics Research Education and Clinical Center, Ann Arbor, MI, United States; ^5^Department of Mechanical Engineering, University of Michigan, Ann Arbor, MI, United States

**Keywords:** near-falls, inertial measurement units, monitoring, accelerometers, balance, walking

## Abstract

**Background:** Near-falls such as a trip, slip, stumble, or misstep involve a loss of balance (LOB) that does not result in a fall, occur more frequently than actual falls, and are associated with an increased fall risk. To date, studies have largely involved detection of simulated laboratory LOBs using wearable devices in young adults. Data on the detection of and kinematics of naturally occurring LOBs in people at high risk of falling are lacking. This may provide a new way to identify older adults at high risk for falls. We aimed to explore key body kinematics underlying real-world trips in at-fall risk community dwelling older adults wearing inertial measurement units (IMU).

**Methods:** Five community-dwelling older adults with a history of falls who reported trips during the study period participated. They wore a voice recorder and 4 IMUs mounted on feet, lower back and wrist for two consecutive weeks to provide a record of the context and timing of LOB events. Sensor data prior to time-stamped voice recording of a trip were processed in order to visually identify unusual foot trajectories and lower back and arm orientations. Then, data of feet, lower back and wrist position and orientation were combined to create a three-dimensional animation representing the estimated body motion during the noted time segments in order to corroborate the occurrence of a trip. Events reported as a trip by the participant and identified as a trip by a researcher, blinded to voice recordings description, were included in the final analysis.

**Results:** A total of 18 trips obtained from five participants were analyzed. Twelve trips occurred at home, three outside and for three the location was not reported. Trips were identified in the sensor data by observing (1) additional peaks to the typical foot velocity signal during swing phase; (2) increased velocity of the contralateral foot and (3) sharp changes in lower back pitch angles.

**Conclusions:** Our approach demonstrates the feasibility of identifying and studying the mechanisms and context underlying trip-related LOBs in at-fall risk older adults during real world activities.

## Background

Falls are a serious problem in older adults, as the leading cause of accidental death as well as hospital admission for injury (1, 2). The ascertainment of risk for falls often relies on patient recall and thus may underestimate the true incidence of falls (3). Moreover, because falls may be precipitated by everyday activities in a specific environmental context, one-time laboratory or clinical assessment of balance and mobility in a highly controlled non-real-world setting may capture important measures of postural control but may not optimally capture real-world fall risk (4, 5). Monitoring real world daily activities using fixed body sensors might therefore enhance fall risk assessment.

Given that falls are relatively rare events requiring very long periods of observation, near falls during daily life activities might then be used to inform fall risk (6). More specifically relevant to the present study are missteps, defined as trips, slips, stumbles and other losses of balance (LOBs) that do not result in a fall because of corrective actions taken to recover balance (7). Self-reported LOBs are more frequent than falls and are linked to fall risk (7–10). In a retrospective observational study of older adults with hip osteoarthritis (*n* = 106, mean age 74.4 ± 6.2) 45% reported at least one fall and 80% reported frequent (i.e., at least once per week) or occasional (i.e., <1 per week but more than a couple times) LOBs over a period of 1 year (8). Nevertheless, using only self-report is limited: it is subjective, relies on memory (and thus may be unreliable in the cognitively impaired) and is subject to reporting fatigue. Using wearable sensor data might provide information when the participant cannot. Moreover, it may provide additional data such as LOB kinematics that cannot be obtained from self-report. Thus, using wearable sensors to identify and quantify LOB kinematics during daily activities might serve as a sensitive measure associated with fall risk (6) but equally important help to characterize the context of the LOB and the strategies used to recover from the LOB.

In controlled settings, wearable sensors can detect artificially induced LOBs with high accuracy (6, 11–15). However, it is thought that LOB responses in controlled settings (such as a laboratory) differ from naturally occurring real world LOB responses (16). Recently, adapting responses from laboratory-induced perturbations, an algorithm was developed to identify real-world missteps (i.e., defined as a loss of balance that would result in a fall if sufficient recovery mechanisms were not activated) (16, 17) in persons with Parkinson's Disease who wore a single inertial measurement unit (IMU) for 3 days (16). Participants with identified fall risk (two falls or more in the 6 months prior to the study) were significantly more likely to have a detected misstep compared with non-fallers. However, without additional corroborative information, these missteps could only be determined as “suspected.” It is thus not yet clear whether wearable devices can accurately detect LOBs in older adults or other high-risk populations while at home or other community settings (15).

We illustrated the methodology to corroborate the occurrence of real-world LOBs in participants wearing an array of IMUs; participants also donned a wearable voice recorder on which they could provide time-stamped descriptions, i.e., the context, of their LOBs (18). In the present paper, we advance the methodology to focus on a specific LOB type in at fall risk older adults. The focus of the current study is on the LOBs that are most clearly identified from the IMU record and corroborated using the voice record: those described as “trips.” Unlike other types of LOBs such as “misstep” and “stumble,” a trip is an identifiable concept in common language representing a unique type of LOBs where the mechanism is clear (i.e., swing leg strikes an obstacle). Yet, unlike laboratory settings, real world trips are unanticipated and may vary in the environment and context where they occur. Whereas, the laboratory-based protocols provide controlled trip perturbations which may result in more stereotypical responses, real world trips may have more variable etiology (i.e., context dependent) and the LOB responses may be equally more variable and less stereotypical. More importantly, better understanding of real world trips would be useful in determining the outcomes of increasingly common reactive balance training protocols (19).

Accordingly, the current study investigates real-world trip responses in at-fall risk community dwelling older adults using wearable IMUs and concurrent participant reporting of the occurrence of a trip. This new methodology may provide insight into trip responses and recovery in at-fall risk older adults and may then be applied to customize an intervention program to reduce fall risk in this population.

## Methods

### Participants

We collected data from 10 community dwelling older adults, all of whom reported LOBs over the study period. Based on this sample we extracted only LOBs that the description obtained from the participant clearly indicated a trip in the recordings (either the participant used the word trip or the described circumstances were consistent with a “trip” e.g., “caught foot on,” “stubbed foot”) resulting in five participants that were included in the analyses reported here. Study inclusion criteria included the ability to ambulate independently in the community and either a history of ≥2 falls in the past 6 months, a history of an injurious fall in the past 6 months, or self-reported balance difficulties. Exclusion criteria included walker use and cognitive deficits (Montreal Cognitive Assessment score <24/30) (20). All participants provided written informed consent before participating in the study. The study was approved by the University of Michigan Institutional Review Board.

### Protocol

#### LOB Monitoring

In order to capture real world trips, participants wore a wrist-mounted voice recorder and 4 body-worn IMUs during waking hours. They were instructed to report any LOB, defined as an event where balance control was lost at least momentarily such as a trip, slip, stumble or misstep, immediately after the event occurred on the voice recording.

### Voice Recorder

The voice recorder was a watch-sized device worn on the non-dominant wrist (Sansa Clip Zip 8GB MP3 player, with modified open source RockBox firmware). The voice recorder was programmed to record for 2 min upon the press of a single button and provide a time stamp for each recording. The recorder clock was synchronized to the clock of IMUs. Participants were asked to record a description of the LOB context (a description of the task performed and any contribution of the environment to the LOB), and how they recovered their balance as soon as possible after the event.

### Wearable IMUs

**Figure 1:**
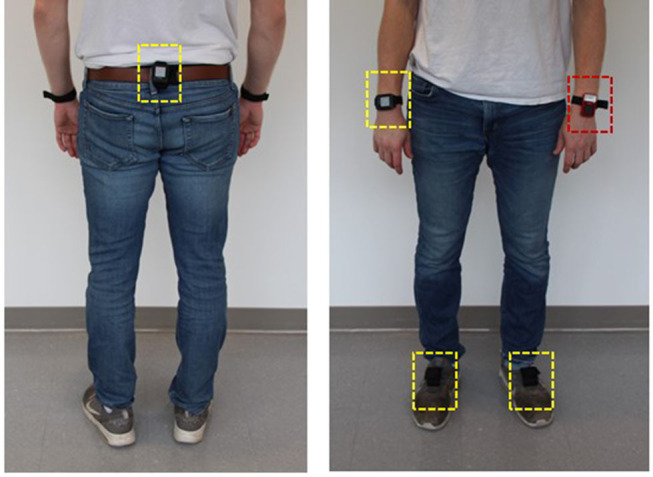
IMUs mounting location at feet, lower back and one wrist (dashed yellow line) and a voice recorder worn on the other wrist (dashed red line).

Participants wore four IMUs (*Opal, APDM Inc., Portland, OR, USA*; synchronized 128Hz sampling, ±16/200G acceleration ±2000 degree/s angular rate) to capture three axes raw acceleration and angular rate. The sensor bandwidth (128 Hz) is adequate for capturing human initiated motion given the low dynamic conditions of our target population (21). The accelerometer range is automatically selected by the data logger as required by the experimental conditions. The IMUs are factory calibrated and we did not perform any additional calibration procedure. Our method does not require following any special anatomical calibration since our method approximates the correct location (see section Data analyses). The IMUs were mounted on the feet using pouches attached to the tops of each shoe, at the center of the lower back on a waist belt and on the wrist (dominant hand) using a wrist strap (Figure 1). Each IMU was marked with a specific location (left foot, right foot, lower back, and wrist). Participants were provided a diagram showing correct sensor insertion configuration. Participants were asked to wear the sensors while they performed their daily normal routine. Participants removed the sensors at the end of the day for overnight charging and inter-IMU resynchronization to the IMUs' clock.

Participants attended two laboratory sessions: On the first session they were instructed on the use of the voice recorder and what to record when they had a loss of balance. They then used the recorder at home for a week. At the second session their recordings were briefly reviewed, and questions were answered. They were instructed on how to wear and charge the IMUs and were provided written instructions to use at home. They then wore the recorder and sensors for two consecutive weeks. Data was collected based on previous experience in which a substantial number of LOBs were reported over a 2 week period.

#### Clinical Assessments

The following clinical measurements were conducted during the first laboratory session: The Timed Up & Go (TUG) test (22, 23) and the Activities-specific Balance Confidence (ABC) scale (24). The TUG assesses mobility, balance and fall risk in older adults. It measures the time that takes the subject to rise from a chair, walk 3 meters at a comfortable pace, turn, walk back to the chair and sit down. The cutoff score indicating risk of falls in community dwelling adults is >13.5 s (23). The ABC scale is a 16-item self-report measure in which participants rate their balance confidence for performing activities (ranges 0–100; score of zero represents no confidence, score of 100 represents complete confidence). ABC scores above 80 are indicative of highly functioning older adults (25).

### Data Analyses

#### Voice Recordings

Voice recordings of LOBs were reviewed by author DS and events described as trips were identified. Time stamps of LOB events were used to focus the analyses of IMU data. A 10-min time window of sensor data prior to the time-stamped event recording was processed using custom developed algorithms written in Matlab (Matworks, Natick MA) (i.e., IMU data were processed only at a defined time window close to the event reported by the participant).

#### IMU Kinematics

**Figure 2:**
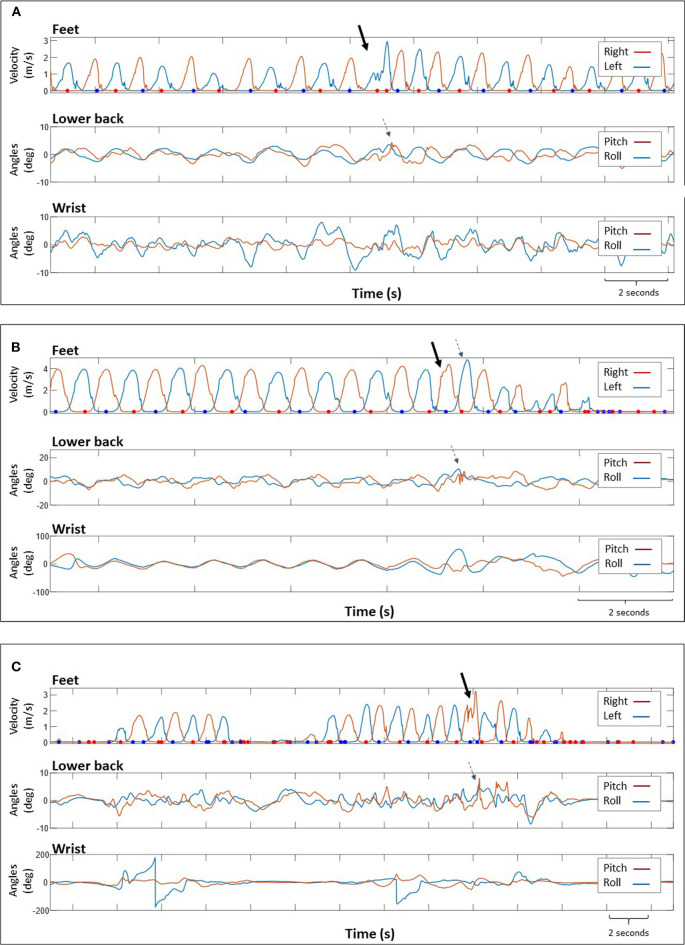
Data obtained from 3 participants, showing: (top) magnitude of foot velocity corresponding to right and left foot and lower back (middle) and wrist (bottom) tilt angles. The dots indicate instances during the stance phase where the foot was stationary. The solid arrows indicate the trip location identified by a sharp increase in velocity or several “peaks” indicating changes in foot velocity. The dashed arrows show the recovery response as a change in velocity of the contralateral foot **(B)** or variations in lower back pitch angles **(A–C)**. We could not identify trips based on data obtained from the wrist sensor. Participant's self-report: **(A)** “Caught left foot under carpet, stumbled” **(B)** “climbing steep hill, tripped over speed bump” **(C)** “Tripped on a piece of wood”.

Raw IMU data (accelerometer and gyroscope) were processed to estimate foot position and orientation. The IMUs measure three components of linear acceleration and angular velocities in the sensor frame. The angular velocities were integrated over time to obtain the IMU orientation. The tilt components (roll and pitch) of the orientation were referenced to gravity using the accelerometer measurements. This method was applied to all four IMUs. For the IMUs attached to the feet, we proceed to resolve the accelerations in the navigation frame using the IMU orientations. The navigation frame referenced accelerations were integrated twice to estimate velocity and position (26, 27). The velocity and positioning estimations are affected by sensor drift, however since the foot comes to a stop during the stance phase of gait cycle, it is possible to use this information to correct the velocities and positions errors using a method known as Zero velocity UPdaTe (ZUPT) (28, 29). From the 10-min time window of IMU data the observer visually identified gait segments using plots of foot speed data (Figure 2). Then the observer sought to identify deviations from the stereotypical pattern of foot trajectories during normal gait (e.g., a sudden change in the rhythmic foot speed during gait, ≥2 steps performed with the same leg, a simultaneous non-zero velocity of both feet at once) that would suggest that an LOB may have occurred. In addition, the lower back and wrist orientations (i.e., roll, pitch, and yaw) were used to identify sharp changes from the usual patterns observed in the vicinity of the event, indicating a compensatory motion.

#### Corroborative Animation

After identifying these unusual patterns (i.e., potential trips), we used the feet, lower back and wrist positions and orientations to create an animation representing the estimated body motion during the time segments. The animation was used for qualitative analysis, rather than estimating the precise multi-segment kinematics, in order to visually corroborate the occurrence of a trip (18). When the animation supported the occurrence of a trip, characteristics features of IMU signals were extracted. For the animation, the lower back orientation was used as a proxy of the trunk motion (30).

We converted the information derived from IMU measurements, taken in the sensor reference frame, into a common reference frame (world). Since the feet, trunk and wrist are interconnected, in the long run they all must follow the approximate same path as the person moves, we combined all this information using a two-step approach. First, we aligned all the position and orientation estimates to follow a common reference coordinate frame. Second, we placed the different body parts relative to the others using anatomical assumptions.

##### Position and orientation alignment

Our approach estimates and corrects the tilt angles (roll and pitch) using gravity as the vertical reference. Because gravity is an absolute reference we conveniently use it as the vertical axis of our world reference frame. The heading angles (yaw) are free to drift, and in the case of the foot-mounted IMUs, the heading errors cause the feet to drift apart in space with time. In order to minimize the effects of drift we implemented transfer alignment techniques similar to the approach explained in (31). This approach employs a Kalman filter framework in which the inertial coordinate frame (world) as defined by a foot mounted IMU, acts as the anchor for all the other IMU estimates of orientation and position. This method guarantees that all the IMU based estimates describe the approximate same trajectory (of one foot), however it does not establish how the IMUs are located with respect to each other.

##### Anatomical alignment

In order to determine the final spatial distribution of the individual IMU based solutions, we use three anatomical assumptions. First we assume a nominal separation between the two feet that is enforced when they cross with respect to each other in the gait cycle, and when the person is standing. The actual correction is applied as part of the same Kalman filter mechanization used to align the orientation and position of all the IMUs as indicated in (31). The second assumption is that the base of the torso is located at a certain distance directly above the mean position of the two feet. The transfer-alignment corrected orientation of the IMU attached to the lower back is used as is to represent the trunk orientation. The third assumption is that the wrist sensor is attached to a forearm connected to a simulated arm at a roughly anatomical elbow location. The transfer-alignment corrected orientation of the IMU attached to the wrist determines the orientation of this simulated forearm. The actual distances used to separate the feet, trunk and forearm follow a similar approach as in (18), and as such are used to provide a qualitative representation of the body motion that can be used for interpretation by a human, rather than to estimate precise multi-segment kinematics.

This process of identifying trips from IMU data was conducted by a researcher (SH) blinded to the voice recordings description of the event. The researcher was given the time segment when the LOB occurred but did not know the type of LOB that was reported. In this paper we report events in which the participant used the word “trip” in his/her description, or described circumstances consistent with a “trip” (e.g., “caught foot on,” “stubbed foot”) AND a trip event was independently identified in the IMU signal analyses. Requiring this agreement between voice and IMU data was done in order to increase certainty levels/face validity of our analysis.

## Results

Characteristics of the 5 participants are shown in Table 1. All participants reported at least one fall within the last 6 months, 2 of them reported an injury as a consequence. Four of the five participants reported ABC scores <80 (and 3 <65), suggesting significant concern in their confidence to maintain balance during everyday activities. Yet, all TUG scores were <14 s, suggesting a functionally mobile group. Thus, while they are at fall risk and have balance concerns, they are nevertheless mobile and likely doing activities that may place them at risk for future trips and falls. Participants wore the IMUs for a mean of 13.2 ± 1.1 days, 11.7 ± 1.7 hours per day. Eighteen events obtained from all of the five participants were documented as tripping from the voice recorder and from the IMUs analysis. From 10 to 15 min were required to visually inspect the IMU data and generate the corresponding 3D animations, depending on the amount of active bouts in the time window being observed. There were no missing data, however there were four trips reported by two participants that were not evident in the IMU analyses (the observer did not identify an LOB during the 10 min epoch in these four cases). Of the 18 events, 12 trips occurred in the home (two on stairs) and three outdoors. For three events the location was not reported. Of these 18 trips, one resulted in a fall onto a bed. Participant descriptions of trips (activity and location) are presented in Table 2.

**Table 1:** Participants' characteristics (*n* = 5).

**Characteristics**	**Values**
Age, years	76.2 ± 5.4
Male (*n*)	4/5
Fall within the past 6 months (*n*)	5/5
Injurious fall within the past 6 months (*n*)	2/5
ABC score (0–100)	64.4 ± 14.0
Use of assistive walking device (*n*)	1/5 (cane)
TUG (s)	11.2 ± 2.1

*ABC, activities-specific balance confidence scale; TUG, timed up & go*.

**Table 2:** Self-reported trip characteristics and sensor signal analysis.

**Participant**	**Description**	**Location**	**Identified trips were based on IMU signals of:**		
			**Tripped foot**	**Contralateral foot**	**Lower back**
1	Carrying trash to curb, caught toe on curb, stumbled	Outside, curb	+	+	–
	Walking to back door to let dog in, stepped on cane	Home	+	+	+
	(see Figure 4)				
	Stubbed toe in middle of floor	Home, kitchen	+	–	–
	Stepped on left foot, stumbled	Home; TV room	+	+	+
	Scuffed foot on floor, toe wouldn't slide	Home, kitchen	+	+	+
	(see Figure 3B)				
	Stubbed toe on floor	Home; kitchen	+	+	–
	Caught left foot under carpet, stumbled	Home; hallway	+	–	+
	(see Figure 2A)				
2	Climbing steep hill, tripped over speed bump	Outside, road	+	+	+
	(see Figure 2B)				
3	Walking, tripped, twisted ankle sideways	Outside, on building construction site	+	–	–
	Stepped in hole, tripped	Unknown	+	+	+
	(see Figure 3C)				
	Tripped on piece of wood	Home; workshop	+	–	+
	(see Figure 2C)				
	Stepped in hole, tripped	Unknown	+	+	–
	Stair descent caught heel	Home, stairs	+	–	+
	Stepped on piece of wood, tripped	Home, barn	+	+	+
4	Tripped	Unknown	+	–	–
5	Tripped on carpet	Home, kitchen	+	–	–
	Caught foot on dresser	Home; bedroom	+	–	–
	Stumbled going upstairs, tripped	Home, stairs	+	–	+
	(see Figure 3A)				

### Sensor Signal Analysis

Trips were identified from feet velocity plots by observing additional peaks in the typical foot velocity signal indicating changes in velocity, particularly abrupt changes during the swing phase, Figure 2. In some cases trips were followed by increased step velocity of the contralateral foot (recovery response) (Figure 2B) and accompanied by sharp changes in lower back pitch angles (Figures 2A–C). Data regarding the location of the sensor (feet and/or lower back) that were used for trip identification are presented in Table 2. We could not identify trips based on data obtained from the wrist mounted sensor as it was difficult to differentiate trip-related changes in signals or derived orientations from normal activity (Figure 2). After identifying a “suspected” trip event from sensor signals, the observer used the animation to corroborate the occurrence of a trip. Representative examples of animations are presented in the Supplementary Materials.

**Figure 3:**
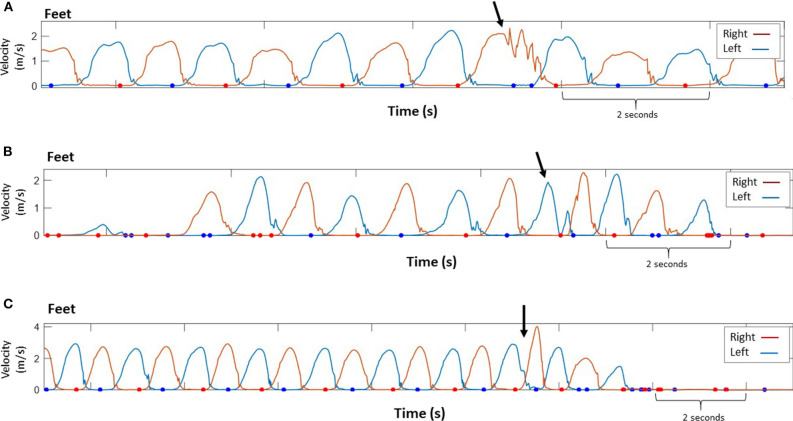
Data demonstrating trips occurring at different times during the swing phase. Magnitude of foot velocity corresponding to right and left foot. The dots indicate instances during the stance phase where the foot was stationary. **(A)** A sudden change in right foot's velocity during mid swing (immediately after peak velocity) while climbing stairs (stereotypical velocity profile of climbing stairs contains short periods of nearly constant velocity). **(B)** A sudden change in velocity of the left foot during mid swing (i.e., the foot almost reached maximal velocity), then the foot landed on the ground (foot velocity equal to zero) and a fast, compensatory step with the same foot was performed. **(C)** A sudden change in maximal velocity of the left foot during terminal swing. An increased velocity of the contralateral (right) foot afterwards. Arrow indicates the trip location. Participant's self-report: **(A)** “Stumbled going upstairs, tripped” **(B)** “scuffed foot on floor, toe wouldn't slide” **(C)** “stepped in hole, tripped”.

**Figure 4:**
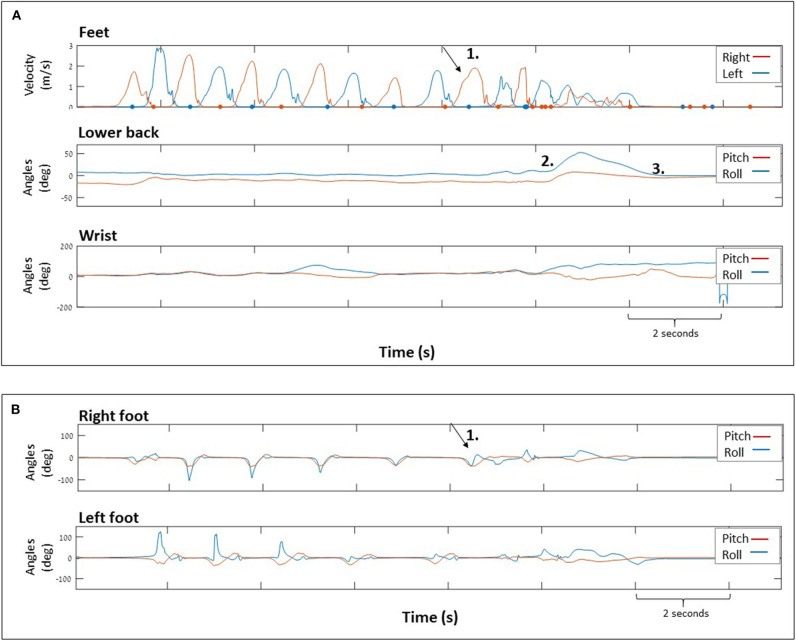
Data showing a trip that resulted in a fall. **(A)** (top) Magnitude of foot velocity corresponding to right and left foot and (middle) lower back and wrist (bottom) tilt angles. Numbers indicating: (1) the trip location (2) lower back moved backwards (increasing pitch angle) and rightwards (increasing roll angle) while the participant fell onto the bed, (3) participant immediately rolled back to mid position (decreasing roll angle) and remained backwards (approximately constant pitch angle). **(B)** (top) right and (bottom) left foot tilt angles. The participant recorded: “Walking to back door to let dog in, stepped on cane. Fell on a bed”.

Figure 3 demonstrates signals indicating trips occurring at different swing phases (early/late swing). Also, trips occurred during varied segments of walking i.e., short segments with only few steps prior to trip vs. long segments of walking with many steps prior to trip. Right and left foot sensor raw data for each trip is represented in the (Figure S1). Figure 4 demonstrates a trip that resulted in a fall onto a bed, i.e., a fall onto a lower surface. In this case, the lower back extended backwards to an inclination angle for a sustained period of time suggesting that upright sitting was not maintained, and given the foot orientations, that the participant was no longer standing (Figure 4).

## Discussion

The present study is the first attempt to our knowledge at identifying and characterizing kinematic parameters of “natural” trips occurring at home and community environments in older adults at risk for falls. We significantly add to previous studies by demonstrating the feasibility of this approach in studying varied real world trip contexts. To date, IMUs have been used to identify LOBs mainly in controlled laboratory settings, however, it is thought that responses in controlled settings differ from naturally occurring real world LOBs. Thus, in order to automatically identify real world LOBs we first needed to examine whether it was feasible to identify them and to establish a method of LOB analytics that might lead to future algorithm development. A major contribution is the use of a voice recorder concurrent with IMU data to identify the context of the LOB and to increase the certainty level of identification of LOBs in older adults. The sensor-based approach supplemented with a voice recorder for description and time stamping of the tripping event used in the current study may considered to be “proof of concept,” that the study of mechanisms and context underlying relevant real world losses of balance is feasible.

In contrast to trips provoked in laboratory settings, where conditions are more controlled and responses are more stereotyped, real world trips are more unanticipated and occur during walking or gait initiation, at different speeds and accelerations, in different environments (e.g., outside, stairs, in the dark) and often concurrent with other tasks (talking, carrying etc.). This lack of homogeneous tripping patterns in the natural setting makes identifying real world trips from IMU data alone, without context information, particularly challenging. Moreover the carryover of reactive balance training protocols into changing real world trip occurrence and response is still unclear.

In the current study, real-life trips were identified from processed IMU data representing feet kinematic information during gait and characterized by additional peaks to the typical foot velocity signal during swing phase (Figure 2). These peaks indicate changes in velocity, particularly abrupt changes during the swing phase. This is consistent with a recent study identifying trips as deviations from a statistical model for normal walking patterns (13). In some cases trip identification was supported by data from the contralateral foot sensor (demonstrating increased velocity of the step taken after the trip) and from the lower back sensor demonstrating a sharp increase in lower back tilt in the pitch axis. This is consistent with a previous study suggesting that near falls may include compensatory mechanisms such as unplanned movement of arms or/and legs, unplanned change in stride velocity and trunk tilt (32). Also, in the current study the timing of a collision with an obstacle during swing phase (early vs. late swing) was identified (Figure 3). Previous studies identified two trip recovery strategies according to the timing of collision with the obstacle: (1) during early swing, collision with an obstacle leads to an elevating strategy, in which the swing leg is lifted over the obstacle and (2) during late swing, collision with an obstacle leads to a lowering strategy, in which the swing leg is rapidly lowered to the ground in front of the obstacle and then the trailing leg is swung forward clearing the obstacle and landing in front of the body (33, 34). Figure 3C shows a collision occurring during late swing, followed by a rapid response (i.e., increased foot velocity) of the contralateral leg.

Identifying kinematic characteristics of a trip is an important step toward automated detection of real-life trips and other naturally occurring LOBs. Previous studies used machine-learning algorithms to automatically detect LOBs generated by young adults acting out different scenarios (e.g., slip, trip, misstep while walking) (11, 12). While the reported sensitivity and specificity was higher than 99% in distinguishing near falls from ADLs (11), the generalizability of these algorithms to detect LOBs in real life situations and in at-fall risk older adults is not clear (15). An algorithm to detect missteps in persons with Parkinson's disease was developed based on laboratory data, however, when the algorithm was applied to daily life conditions, events identified by the algorithm were labeled as “suspected missteps” due to the lack of information about context (16). We believe that the proposed approach that incorporates a voice recorder for time stamping of the tripping event with IMUs data (signals and animation) improves the certainty level of identifying trips occurring in real life. The current study is only the first step toward an algorithm for automated trip detection. An automated detection of real world data with greater variability in the trip etiology/context and then subsequent response presents a major challenge. The fact that all trips were identified from feet velocities during swing phase and that we could visually identify them supports the notion that it may be feasible. Future studies applying similar methods are needed to acquire a sufficiently large data set of real-world trips in older adults at risk for falls. Then, an algorithm for automated trip detection using IMU data might obviate the need of a voice recorder to provide a time stamp.

Even as these algorithms are developed, voice recorder information still adds valuable information regarding trip context (as is presented in Table 2). A previous study analyzing real-life falls captured on video in long-term care facilities revealed that 25% of trips occurred due to the foot being caught on a chair or table leg (i.e., causing a trip), thus suggesting the need for improved staff awareness of this hazard, and improvements in environmental planning and furniture design (35). The context of the trip which includes the environment (home/outside), specific location (curb, stairs etc.) the time (morning/night) in addition to data regarding the amount of trips, the leg that tripped and the timing of the trip during swing phase may be important clinically for a personalized fall prevention intervention. For example, home modifications may be important for a person who trips at the same location in house. A targeted exercise program may be useful for a person who repeatedly trips with the same leg. Should trips be detected automatically in the future, the voice recorder would not be needed for the time stamp function but may still play a role in providing vital information regarding tripping context.

As a first step in characterizing real world LOBs, we focused on trips because they are an identifiable concept in common language and represent a unique type of LOBs where the mechanism is clear. In order to increase the face validity, we restricted ourselves to analyze only events that were reported as trips by the participant and by an observer blinded to participant's self-report. Further study is needed to identify and characterize other types of LOBs such as “missteps” or “stumbles,” broader terms that might suggest LOBs caused by different mechanisms, thereby resulting in different kinematic characteristics.

While in the current study most trips have been recovered (17/18 trips), one trip resulted in a fall onto a bed. An important characteristic that distinguished this trip from trips that were recovered was the fact that during the fall, the lower back and the foot orientations were in an inclination angle for a sustained period of time indicating that the participant was no longer standing. Although it is possible that the participant might have chosen to get in such body configuration intentionally (i.e., when going to bed), the time that it took to get into that condition was very short and the participant rapidly returned to an upright position (Figure 4).

Regarding the number of sensors and body locations needed to identify LOBs, previous studies have demonstrated the feasibility of LOBs detection using one sensor (6, 12, 13). However, these LOBs were induced in laboratory settings and may not provide sufficient data to detect real world LOBs. Adding more sensors for the detection of real-world LOBs might improve detection accuracy. Here we used four sensors attached to the feet, lower back and wrist. We found that identifying trips was based mainly on data obtained from feet and lower back, which could lead to future studies with fewer IMUs worn. This is in line with a previous study demonstrating that in comparison to other sensor locations, feet kinematics were essential to identify near-falls (11).

Several limitations of the study should be acknowledged. First, our sample size was small, and restricted to independent living, older adults at higher fall risk. Yet, it demonstrates the feasibility of our approach. Further study with larger sample sizes and other at-fall risk populations is needed. Second, most trips did not result in falls. The extent to which the observed signals would persist had the LOBs resulted in falls is not clear although we would expect that the accelerations related to a fall would be even greater following the initial trip. Previous studies (7–10) link losses of balance to eventual falls, and the methods provided here can lead to an estimate of fall risk. Furthermore, while trips do not account for all falls, they (1) represent an important cause of falls (36), and (2) are the target for an emerging area of training (perturbation training) that has been found to reduce falls (37). Thus, study of the processes underlying real world trip LOBs and recovery is important. Third, in order to increase certainty level of analyzing trips (and not other LOB types), we analyzed only events that were reported as trips by the participant and that were classified as trips in review of IMU signals. However, there were four cases that were reported as trips by the participants that were not evident in the IMU analyses. These cases might represent trips that were outside the viewing epoch because the participant delayed recording the event. In one case, there were no walking segments during the 10 min epoch, thus increasing our suspicion that the participant delayed recording the event. The IMUs are sensitive to capture even small kinematic changes, however LOBs that produce little overall motion variation may still go undetected as they could be considered normal motion by the observer. Fourth, we acknowledge that our approach depends on identification of the LOB using foot velocities; given that the focus of our study was on trips, this signal was the most likely indicator that a trip may have occurred. We believe that our approach is still valid with other foot initiated LOBs such as slips, and even trunk initiated LOBs that require compensatory foot motion (e.g., fast abnormal steps). However, we recognize that our method has limitations in other LOB cases that produce little or no foot motion variation such as losing balance while trying to stand. The fifth limitation is the reliance on visual inspection of the IMU data for the trips. This reliance may be reduced dramatically in the future by algorithms for automatic trip detection. This would also require testing the algorithm's sensitivity and specificity in distinguishing trips from activities of daily living (ADLs). Nevertheless, there may also be a need in the future for at least some visual confirmation of automatically detected trips. Furthermore, it is unlikely that these algorithms can adequately substitute for the rich context information provided by the voice recording.

## Conclusions

Our approach demonstrates the feasibility of identifying and studying the mechanisms and context underlying trip-related LOBs in at-fall risk older adults during real-world activities. To our knowledge this is the first study seeking to identify real-world trips in at-fall risk older adults. Trips were identified from feet and lower back IMUs, indicating abrupt deviations from the typical foot velocity signal during swing phase (additional peaks) and in lower back pitch angles. Our long-term goal is to increase the sample size and develop an algorithm for automated detection of real-world LOBs and trips in particular. Better understanding of real world trips would be useful in determining the outcomes of reactive balance training protocols (19, 37). These data can also provide insight into the frequency, type and characteristics of real world LOBs and recovery and might then provide a more precise estimate of fall risk and lead to more customized treatment for fall prevention.

## Data Availability Statement

The datasets used and/or analyzed during the current study are available from the corresponding author on request.

## Ethics Statement

The studies involving human participants were reviewed and approved by the University of Michigan Institutional Review Board (HUM00073568). The patients/participants provided their written informed consent to participate in this study. Written, informed consent was obtained from the individuals for the publication of any potentially identifiable images or data included in this article.

## Author Contributions

NA, LN, DS, and LO conceived and designed the experiments. LN, DS, and NM performed the experiments. SH, NM, and LO analyzed the data. SH, LN, DS, and NM performed data collection. SH, NA, LN, DS, and LO drafted the manuscript. SH, LO, and NA wrote the paper. SH, NA, LN, DS, NM, and LO participated in data interpretation and writing (and approving) the final version of the manuscript. All authors contributed to the article and approved the submitted version.

## Conflict of Interest

The authors declare that the research was conducted in the absence of any commercial or financial relationships that could be construed as a potential conflict of interest.

## Supplementary Material

Representative examples of animations used to corroborate the occurrence of trips. The animation was viewed using a motion animation visualizer [Mokka, MOtion Kinematic & Kinetic Analyzer, Biomechanical ToolKit (BTK)].

Video S1 Demonstrating a trip while walking. Left foot (blue) tripped at 534.89 s (time is presented at the bottom left) IMU data for this video are presented in Figure 2A. Participant's description: “Caught foot under carpet, stumbled”.

Video S2 Demonstrating a trip while climbing stairs. Right foot (green) tripped at 592.24 s (time is presented at the bottom left) (IMU data for this video are presented in Figure 3A). Participant's description: “Stumbled going upstairs, tripped”.

Figure S1 Data demonstrating trips occurring at different swing phases. **(A1)** A sudden change in right foot's velocity during mid swing (immediately after peak velocity) while climbing stairs (stereotypical velocity profile of climbing stairs contains short periods of nearly constant velocity); **(A2)** Right foot sensor raw data; **(A3)** Left foot sensor raw data; **(B1)** a sudden change in velocity of the left foot during mid swing (i.e., the foot almost reached maximal velocity), then the foot landed on the ground (foot velocity equal to zero) and a fast, compensatory step with the same foot was performed; **(B2)** Right foot sensor raw data; **(B3)** Left foot sensor raw data; **(C1)** a sudden change in maximal velocity of the left foot during terminal swing. An increased velocity of the contralateral (right) foot afterwards; **(C2)** Right foot sensor raw data; **(C3)** Left foot sensor raw data. **(A1, B1, C1)** Magnitude of foot velocity corresponding to right and left foot. The dots indicate instances during the stance phase where the foot was stationary. Arrow indicates the trip location. Participant's self-report: **(A)** “Stumbled going upstairs, tripped”; **(B)** “scuffed foot on floor, toe wouldn't slide”; **(C)** “stepped in hole, tripped”.

## Abbreviations

LOB: loss of balance
IMU: inertial measurement unit.
