# Supplementary figures and images for: Detection of Real-World Trips in At-Fall Risk Community Dwelling Older Adults Using Wearable Sensors
Source: Front Med (Lausanne). 2020 Sep 2;7:514. doi: 10.3389/fmed.2020.00514 (PMC7492551; doi:10.3389/fmed.2020.00514)

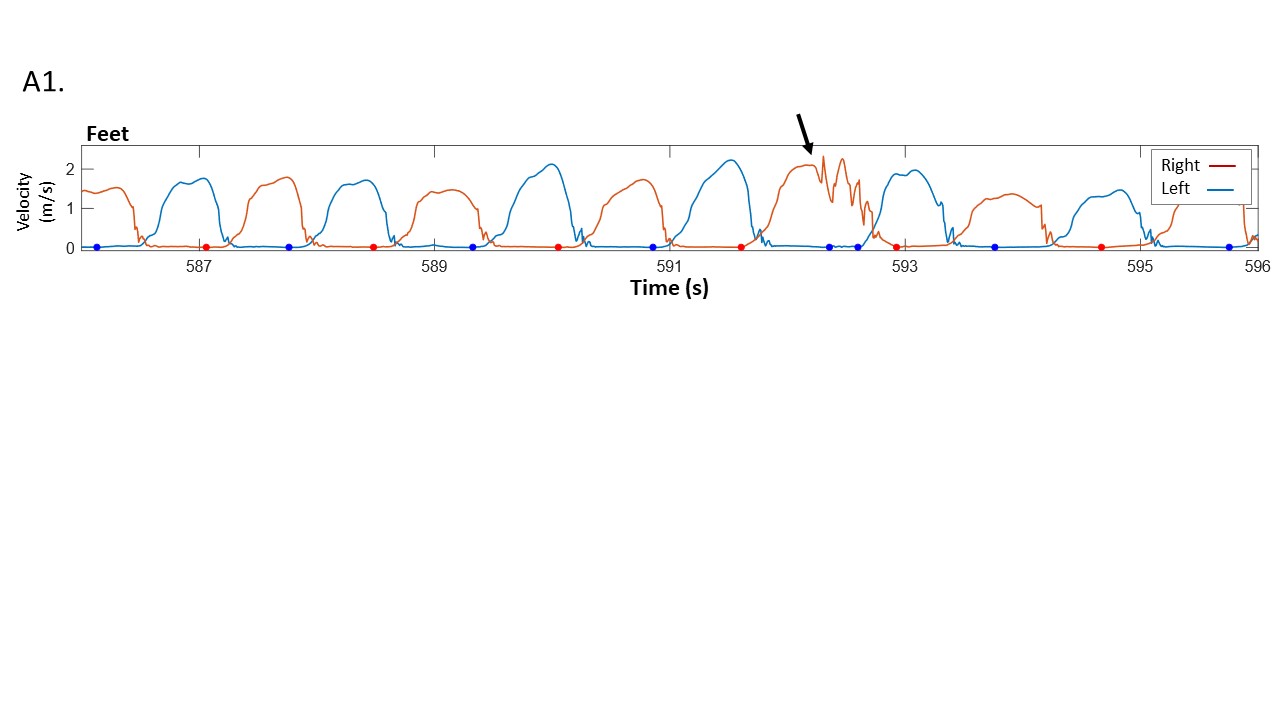

Supplement: Supplementary file 3 [file Image_1.JPEG]

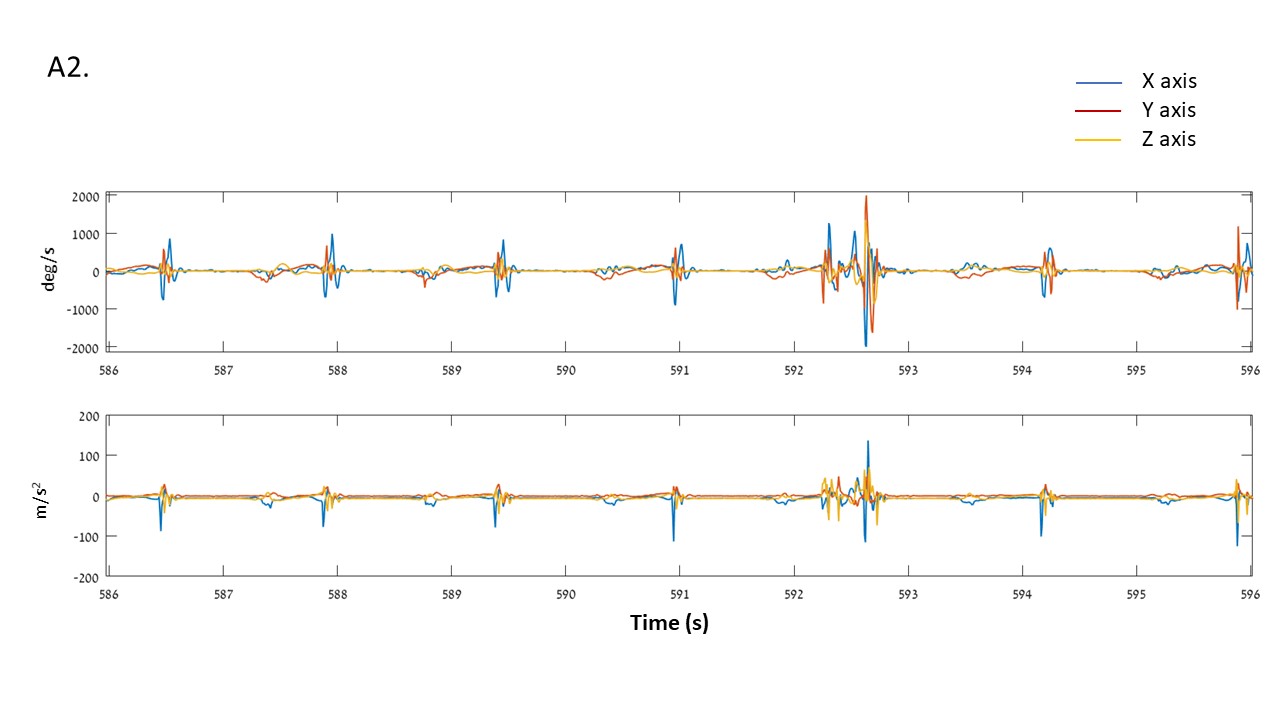

Supplement: Supplementary file 4 [file Image_2.JPEG]

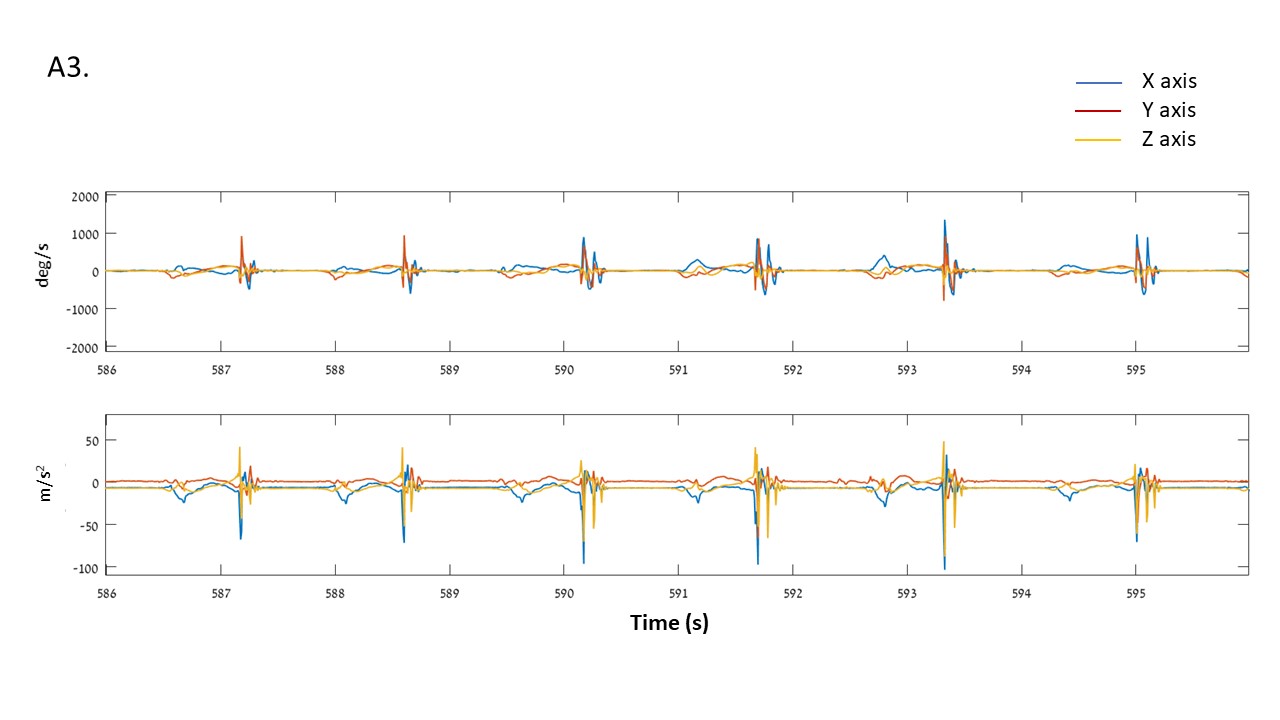

Supplement: Supplementary file 5 [file Image_3.JPEG]

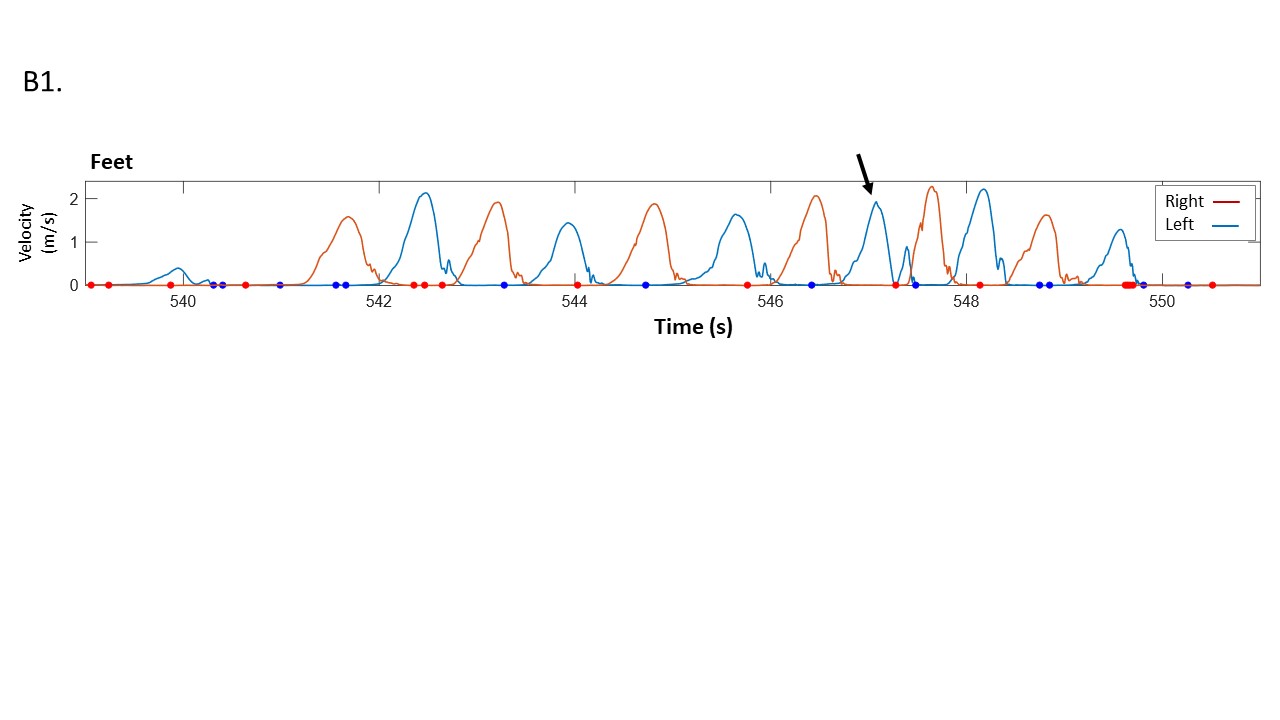

Supplement: Supplementary file 6 [file Image_4.JPEG]

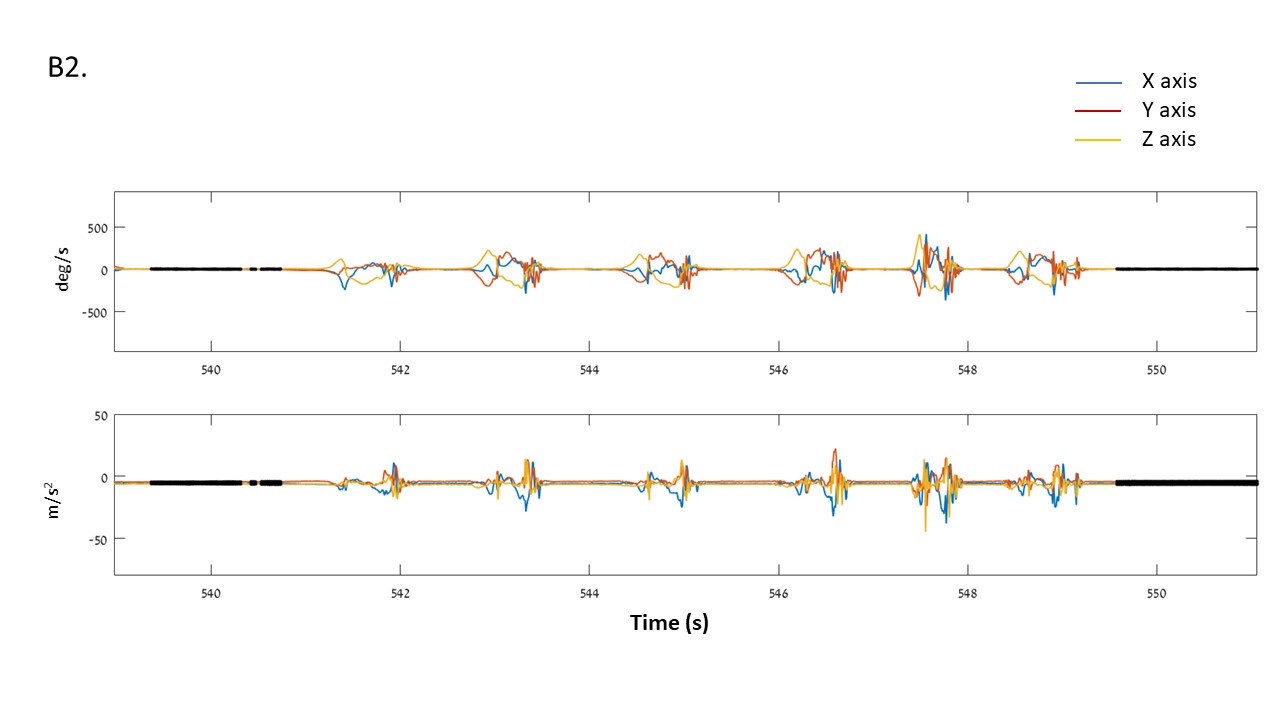

Supplement: Supplementary file 7 [file Image_5.JPEG]

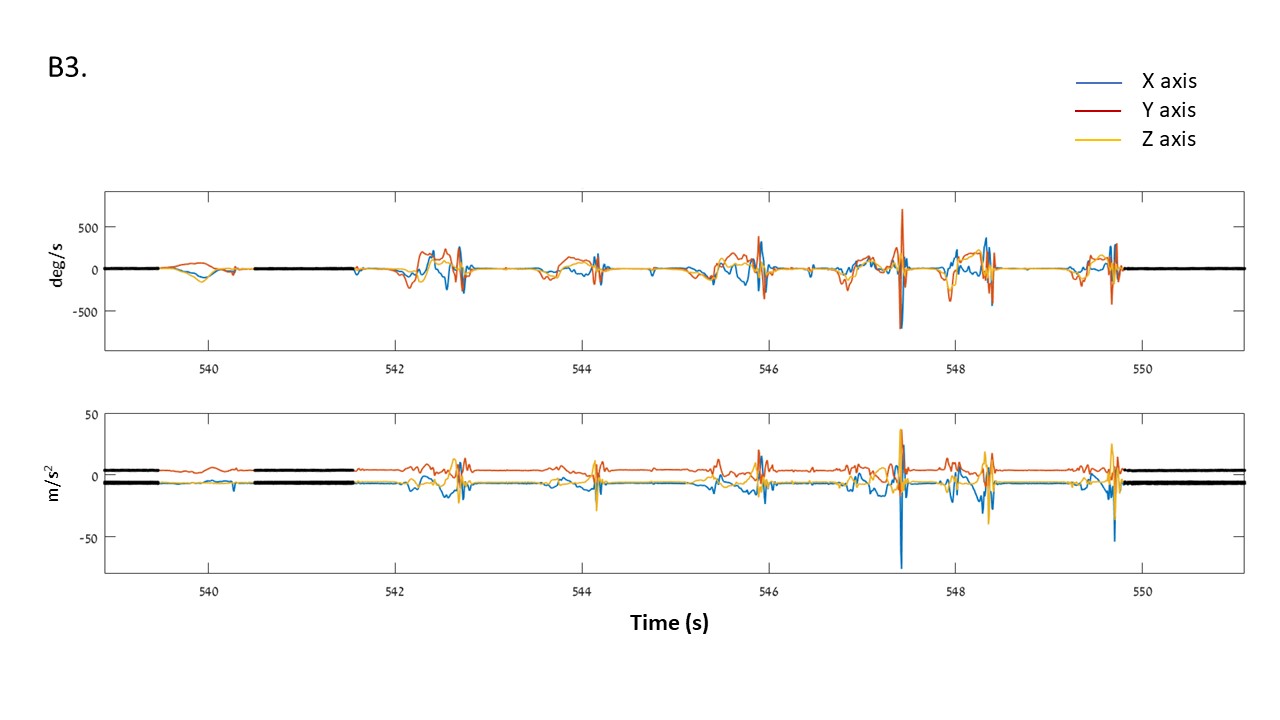

Supplement: Supplementary file 8 [file Image_6.JPEG]

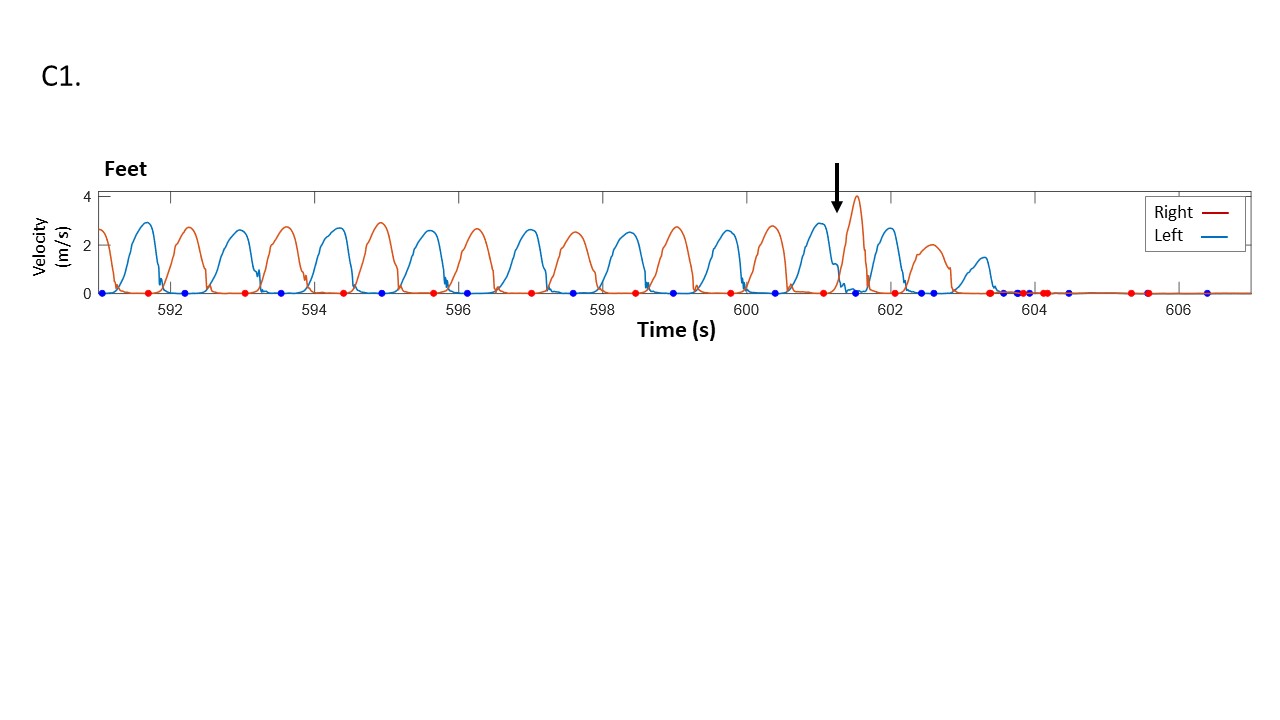

Supplement: Supplementary file 9 [file Image_7.JPEG]

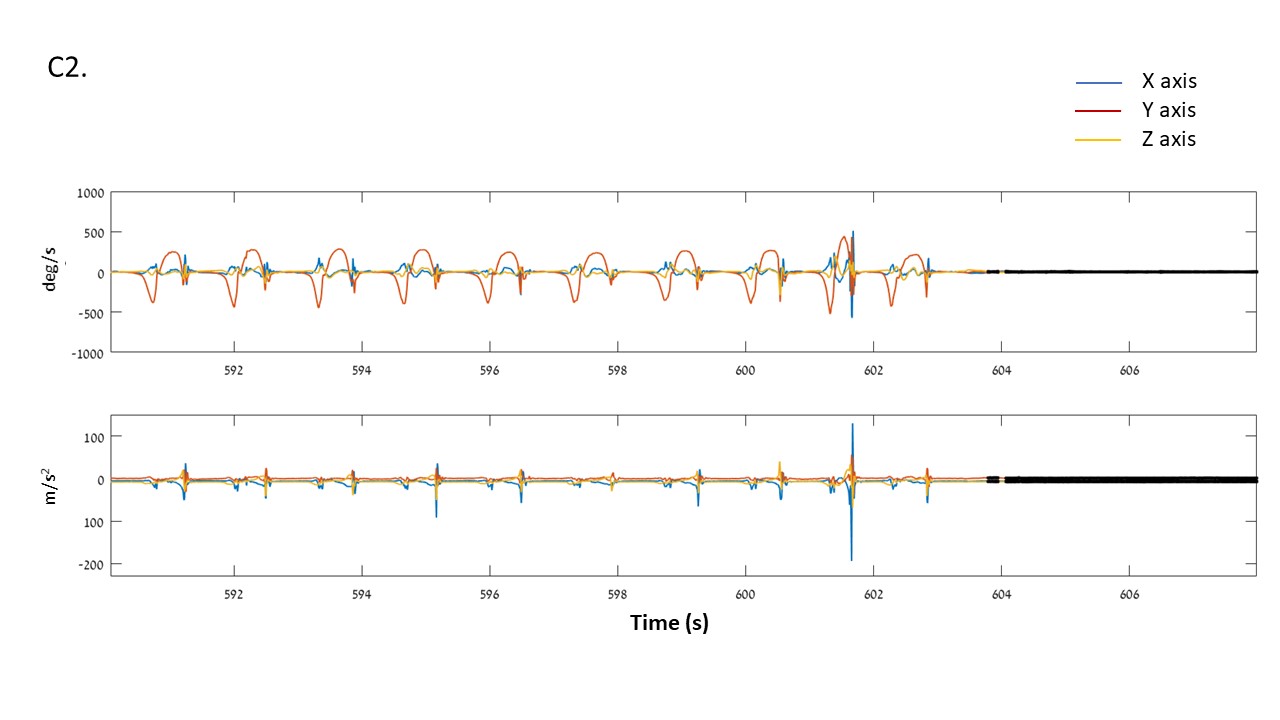

Supplement: Supplementary file 10 [file Image_8.JPEG]

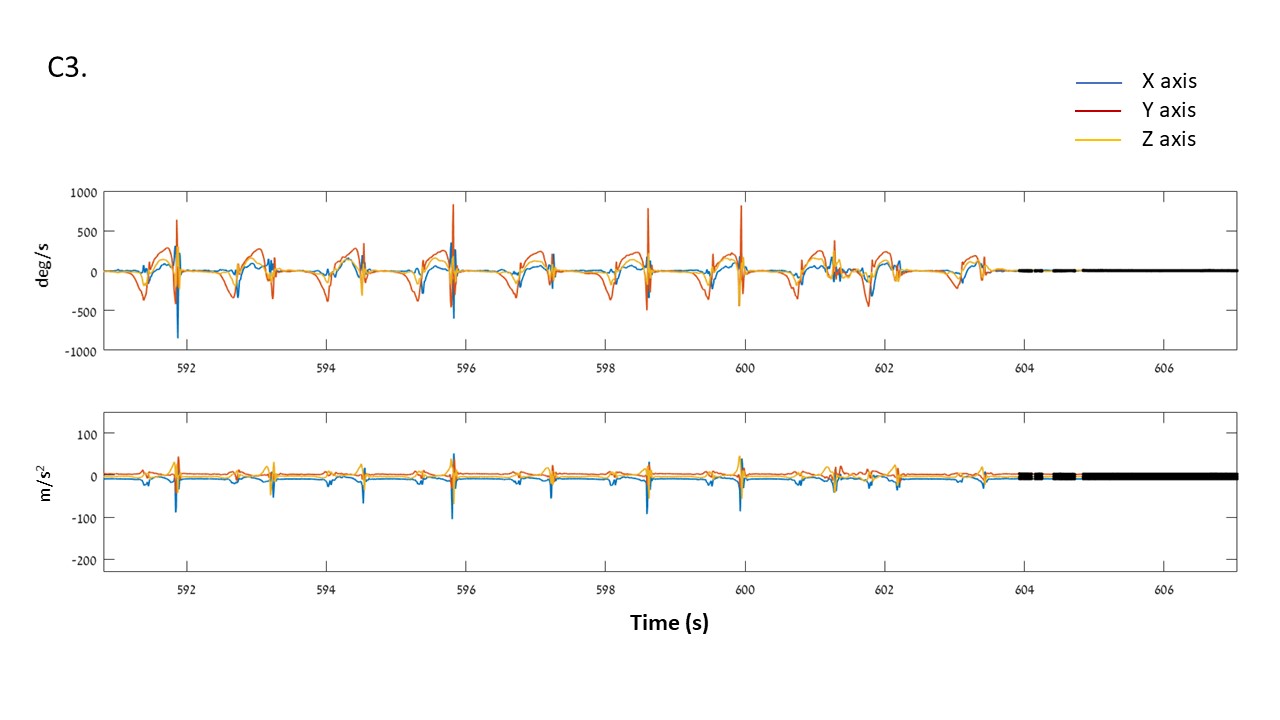

Supplement: Figure S1 — Data demonstrating trips occurring at different swing phases. (A1) A sudden change in right foot's velocity during mid swing (immediately after peak velocity) while climbing stairs (stereotypical velocity profile of climbing stairs contains short periods of nearly constant velocity); (A2) Right foot sensor raw data; (A3) Left foot sensor raw data; (B1) a sudden change in velocity of the left foot during mid swing (i.e., the foot almost reached maximal velocity), then the foot landed on the ground (foot velocity equal to zero) and a fast, compensatory step with the same foot was performed; (B2) Right foot sensor raw data; (B3) Left foot sensor raw data; (C1) a sudden change in maximal velocity of the left foot during terminal swing. An increased velocity of the contralateral (right) foot afterwards; (C2) Right foot sensor raw data; (C3) Left foot sensor raw data. (A1, B1, C1) Magnitude of foot velocity corresponding to right and left foot. The dots indicate instances during the stance phase where the foot was stationary. Arrow indicates the trip location. Participant's self-report: (A) “Stumbled going upstairs, tripped”; (B) “scuffed foot on floor, toe wouldn't slide”; (C) “stepped in hole, tripped”. [file Image_9.JPEG]
